# Diabetes quality of life, Chemical Intolerance, and generational status in a Latino sample: an RRNeT study

**DOI:** 10.1007/s40200-023-01374-3

**Published:** 2024-02-14

**Authors:** Yajaira Johnson-Esparza, Robert Wood, Inez Cruz, Raymond Palmer

**Affiliations:** Department of Family & Community Medicine, University of Texas Health Science Center at San Antonio, 7703 Floyd Curl Drive, MC 7843, San Antonio, TX 78229 USA

**Keywords:** Chemical intolerance, Chemical sensitivity, Diabetes distress, Type 2 diabetes, BREESI

## Abstract

**Objectives:**

The impact of diabetes on quality of life (QoL) includes deficits in physical functioning and emotional and mental health. Individuals with diabetes can experience significant distress related to diabetes management and burden. Comorbid conditions can contribute to QoL among individuals with diabetes. Chemical Intolerance (CI) has received minimal attention in the context of diabetes. CI is characterized by multi-systemic symptoms initiated by a one-time high dose or persistent low-dose exposure to environmental toxins. Latinos experience diabetes distress and are exposed to environmental factors at disproportionate rates. This study sought to investigate generational differences in diabetes QoL and CI in a Latino sample across generational status.

**Methods:**

To assess the modifying effect of CI between QoL and generations, the analysis was stratified by those with and without CI.

**Results:**

Compared to 2nd generation Latinos, Latino immigrants were more likely to indicate that without diabetes, their life would be better across several QoL domains. Latino immigrants had a lower A1C and BMI than2nd generation Latinos. Additionally, they were less likely to have scores indicative of CI than U.S.-born Latinos. QoL varied by generational status and suggested a greater emphasis on family and social relationships among immigrants than U.S.-born Latinos. There were no differences in health services measures across generations.

**Conclusions:**

This study highlights generational differences in the impact of diabetes on QoL. It is one of the few studies to seek to understand the impact of CI on QoL in the context of a chronic condition such as type 2 diabetes.

## Background

Diabetes is the seventh leading cause of death in the United States and the leading cause of kidney failure, lower-limb amputations, and adult blindness [1]. As of 2020, 11% of Americans have a diagnosis of type 2 diabetes [1]. Prevalence of diabetes is higher among non-Latino Blacks (12.1%) and Latinos (11.8%) as compared to non-Latino Whites (7.4%) [2]. The impact of diabetes on quality of life (QoL) includes deficits in physical functioning, emotional and mental health, and body pain [3]. Moreover, diabetes-related distress, includes the management of medicines and behavioral regimens which can be difficult for some, leading to lower QoL [4]. People of color are more likely to experience diabetes-related distress [5, 6] and poor QoL [7, 8] than non-Latino Whites due to multiple social determinants of health, including food insecurity, perceived discrimination, neighborhood, and financial and housing instability, among others.

Chemical Intolerance (CI) is characterized by multi-systemic symptoms (e.g., cognitive, affective, musculoskeletal, gastrointestinal, genitourinary, and cardiovascular) initiated by a one-time high dose or persistent low-dose exposure to environmental toxins. New-onset intolerances often occur when an individual is subsequently exposed to structurally unrelated chemicals, foods, and/or drugs [9] The prevalence of CI ranges from 2 to 13% in non-clinical populations [10–12] and as high as 20% in clinical populations [13]. A recent study found that individuals with CI had elevated glucose and insulin levels, higher long-term blood glucose (i.e., HbA1C), and were more insulin resistant than a control sample [14]. To our knowledge, this is the only study that has considered the relationship between CI and a metabolic disorder, such as diabetes. This is an important area of research given that CI is associated with significant impairment in social and occupational functioning [15–17]. One study found that almost 14% of respondents lost employment due to CI [15], whereas another study found that over half of participants had to leave their work due to CI [16]. Additionally, relationships with extended and immediate family and friends are increasingly affected as the severity of CI increases [15].

Considering the literature demonstrating that comorbid conditions contribute to QoL among individuals with diabetes [17], it may also be important to consider the role of CI on QoL among individuals with diabetes. Jing and colleagues conducted a systematic review of the literature on factors associated with QoL of individuals with type 2 diabetes and found that hypertension and depression were associated with worse QoL [18]. Similarly, in a review of QoL in patients with diabetes in a primary care setting, Galvez Galvan and colleagues found chronic comorbidities and complications were inversely associated with health related QoL [17]. However, there is a gap in the literature investigating the role of the impact of CI on the QoL among individuals with type 2 diabetes.

Moreover, type 2 diabetes QoL may disproportionately impact individuals who are also at increased risk of developing CI. Low-income and communities of color are disproportionately exposed to pollutants and other environmental hazards [19, 20]. placing them at greater risk of or exacerbation of existing CI symptoms. Large quantity hazardous waste generators and proposed Superfund sites^1^ are most prevalent in U.S. counties with a greater concentration of immigrant and non-English speaking households [21]. Exposure to such pollutants have been shown to be associated with an increased risk of illnesses, including poor cognitive and behavioral outcomes [22], respiratory illnesses [23], and cancer [24, 25].

Immigrants may be at particular risk, given the greater likelihood of living below the poverty line as compared to their U.S.-born counterparts. Additionally, immigrants of certain racial/ethnic backgrounds (e.g., Latino and Asian) are more likely to have occupations that expose them to solvents, cleaning agents, pesticides, and other substances linked with the development of CI [26]. Immigrants are in a particularly unique situation, given that with migration, comes exposure to a multitude of stimuli that can lead to increased sensitivity and intolerance. In addition to new pollutants and chemicals, immigrants are also exposed to new allergens, climate, foods, and housing conditions, all of which can impact their physiological response. It is also often the case that manifestation of symptoms associated with exposure (e.g., allergic reactions) increase with years of residence in the host country [27].

The purpose of this study is to investigate generational differences in diabetes QoL and CI in a sample of Hispanic/Latino participants. The association between generational status and medical service use will also be examined.

Although this was an exploratory study, the authors made the following provisional hypotheses: (1) poorer diabetes QoL will be more pronounced among 1st generation individuals. (2) CI will moderate the association between generation and QoL—being more pronounced among first generation relative to U.S.-born counterparts. (3) Medical service use will be highest among first generation relative to others.

## Methods

### Participants and sampling

Patients with type 2 diabetes receiving routine outpatient care from a primary care physician at a residency clinic participating in the Residency Research Network of Texas (RRNeT) were asked to complete a series of surveys on social determinants of health, diabetes QoL, diabetes distress, and other key health outcomes related to diabetes management. RRNeT is a practice-based research network consisting of 11 family medicine residencies and academic health centers across Texas. Inclusion criteria for this study were a diagnosis of type 2 diabetes and being between the ages of 18–75. Participants with cognitive deficits were excluded from the study. Patients who met inclusion criteria and were willing to participate were consented into the study and advised of the voluntary nature of the studyM. Data collection lasted approximately six months. This was a cross sectional descriptive study. This research was reviewed and approved by the University of Texas San Antonio Health Sciences Center Institutional Review Board (IRB; protocol number HSC20180225H).

### Measures

Race, ethnicity, age, and gender were all self-reported, allowing individuals to disclose iformation they felt comfortable sharing. Participants indicated their generational status: 1st generation (born in another country, not the USA), 2nd generation (born in USA, one or both parents born in another country), and 3rd generation (born in USA, both parents born in USA, all grandparents born in another country). Regarding income, participants reported total combined household income in the past month (included employment disability, child support, TANF, student loans).

CI was assessed using the Brief Environmental Exposure and Sensitivity Inventory (BREESI) [12, 28, 29]. The BREESI is derived from the Quick Environmental Exposure and Sensitivity Inventory (QEESI) [30, 31], a 50-item validated questionnaire designed to identify individuals with CI. Like the QEESI. The BREESI is self-administered and assesses an individual’s tendency to react adversely to diverse substances. The BREESI can be administered in less than a minute and consists of three questions pertaining to chemical, food, and drug intolerances. The BREESI has excellent positive and negative predictive validity (97% and 95%, respectively), as well as sensitivity and specificity (90% and 87%, respectively). A sum score of the three items is an accurate screen for CI [12, 28, 29].

Quality of life items for this study were guided by the initial development of the Audit of Diabetes Dependent Quality of Life-13 (ADDQoL-13) [32]. The ADDQoL-13, which has since been replaced by the ADDQoL-18, was a self-administered questionnaire that taps into 13 life domains affected by diabetes: employment, social life, family relationships, friendships, sex life, leisure opportunities, travel, worries about personal future, worries about one’s family’s future, motivation, physical activities, people’s reactions, enjoyment of food. Respondents are asked to consider how their life would be affected (e.g., better or worse) if they did not have diabetes. Responses range from − 3 to 3 (e.g., a great deal better – a great deal worse). Additionally, there are two items that serve as single-item indicators of QoL and impact of diabetes on QoL (“In general, my present quality of life is…” and “If I did not have diabetes, my quality of life would be…”). The AADQoL-13 demonstrated high internal consistency (Cronbach’s alpha 0.85), For purposes of this study, the AADQoL-13 was modified and only the following items were included to limit burden on the respondent: employment, worries about personal future, social life, family relationships, friendships, sex life, worries about one’s family’s future, motivation, and physical activity, as well as the two single-item indicators. Additionally, the range of responses was modified from − 3 to 3 to 1 to 5 for the sake of simplicity.

A modified version of the Diabetes Distress Scale-17 (DDS-17) was used to assess diabetes distress. The DDS-17 is 17-item self-report instrument assessing psychosocial distress due to diabetes across four domains: emotional burden, physician distress, regimen distress, and interpersonal distress [33, 34]. A total distress score is also obtained. The DDS-17 uses a Likert scale to score each item from 1 (no problem) to 6 (serious problem) during the last month. The DDS-17 demonstrates adequate internal consistency (Cronbach’s alpha 0.88–0.93). For purposes of this study, regimen distress was omitted to reduce participant burden. Items to this domain were specifically omitted given the social and medical complexity of this population and the expectation that regiment distress would be elevated.

Health Services measures and A1C were directly obtained from medical records by medical students at participating sites.

### Statistical analysis

Initial analyses consisted of obtaining descriptive statistics and inspecting data for outliers (values > than 3 standard deviations from the mean). Cross-generational comparisons over the QoL and Health Services measures were obtained using a general linear model (ANOVA) with Tukey adjustment to control for Type I comparison-wise error rate.

To assess the modifying effect of CI between QoL items and generations, the analysis was stratified by those with and without CI. We have previously demonstrated the high probability (95%) of being classified as chemically intolerant for those choosing 2 or 3 BREESI items [12, 28, 29]. In this analysis, the sample was stratified by those choosing 2 or 3 BREESI items (Chemically Intolerant group), and those choosing 0 or 1 (Not Chemically intolerant).

## Results

A total of 627 individuals participated in the larger study investigating the impact of social determinants of health on diabetes management and were eligible for the present study. This sample size yields 88% power to detect a moderately small effect size (Cohen D = 0.17). Power analysis was determined for ANOVA. Approximately 60% identified as female and the mean age was 59.6 (*SD* = 12.8). Of the total study sample, 286 (45.6%) identified as White, 150 (23.9%) as Black/African American, 16 (2.6%) as Asian, 11 (1.8%) as American Indian/Alaska Native, 97 (15.5%) as “other”, and 67 (10.7%) did not disclose or chose not to answer. Additionally, 283 (45.1%) participants identified as Hispanic/Latino. Most participants reported being monolingual English-speaking (*n* = 350, 55.9%), whereas 209 (33.3%) reported being bilingual (English, Spanish), 42 (6.7%) reported being monolingual Spanish-speaking, and 10 (1.6%) reported speaking another language. There were 19.6% first generation individuals, 17.5% second generation, and 62.2% third generation. The issue of race and diabetes QoL is addressed in another paper from this data. Due to insufficient numbers of 1st and 2nd generation participants among White (4.2%) and Black (5.8%), we focused on the Hispanic/Latino sample where there were 28% first and 27% 2nd generations.

Table 1 shows the distributions of the sample by generational status. The first generation is statistically older than the other generations (*p* < .04) with no generational differences in the distribution of gender. First-generation individuals generally have lower incomes, less education, and are less likely to have private insurance than 2nd or 3rd generations. The distribution of BREESI items (total sum of three, the individual items themselves, and those classified as chemically intolerant) did not differ across generations. We estimate that the overall prevalence of CI in this sample is 27.5% among this Latino sample.

**Table 1:** Sample demographics across generational status

Demographics/Variable	1st generation n = 72	2nd generation n = 68	3rd generation n = 116
Mean age (SD) *	61.0 (12.0)	55.4 (13.2)	58.1 (12.7)
Percent female	66.2%	58.5%	69.6%
Income <$1000	40.4%	36.7%	29.6%
$1001-$2000	5.3%	20.0%	21.3%
$2001-$3000	36.8%	20.0%	29.6%
>$3000 *	17.5%	23.3%	19.4%
Education			
Less than High School	53.6%	20.6%	20.7%
High School	24.6%	36.8%	38.8%
Some College	2.9%	25.0%	22.4%
Junior college/vocational	8.7%	5.9%	9.5%
College, Graduate/Professional School *	10.1%	11.8%	8.6%
Insurance			
None	5.6%	3.1%	2.6%
Government	79.2%	53.0%	63.8%
(Medicare/Medicaid) Private *	15.3%	43.9%	33.6%
Number of total BREESI items endorsed			
Mean (SD)	0.94 (0.87)	0.89(1.01)	1.10 (0.95)
% BREESI Chem	49.3%	41.9%	54.1%
% BREESI Foods	11.8%	14.5%	19.8%
% BREESI Drugs	33.3%	32.3%	36.4%
Chemical Intolerance:			
Two or more BREESI items	26.1%	25.8%	30.6%
Less than two BREESI items	73.9%	74.2%	69.4%

* *p* < .05

Table 2 shows the Quality-of-Life measures across Generational Status. Relative to the 2nd generation, first generation participants scored statistically lower on the QoL items concerning family relationships, worries about the future of family and close friends, their sex lives, their motivation to achieve, and diabetes quality of life. According to the valance of the 1–5 scale, lower scores indicate that without diabetes, their life would be better in these QoL domains.

**Table 2:** Quality of Life across generational status

Variable	1st generation	2nd generation	3rd generation
	Mean (SD)	Mean (SD)	Mean (SD)
In general, my quality of life is: 1 = very good, 5 = very bad	2.11 (0.75)^a^	1.91 (0.81)^a^	2.00 (0.77)^a^
If I did not have diabetes:			
My employment/career opportunities would be: 1 = great deal better, 5 = great deal worse	2.09 (0.9)^a^	2.32 (0.86)^a^	2.3 (0.85)^a^
My worries about my future (e.g., health, independence, income) would: 1 = decrease a great deal, 5 = increase a great deal	2.28 (1.07)^a^	2.42 (0.99)^a^	2.47 (0.99)^a^
My social life would be: 1 = great deal better, 5 = great deal worse	2.19 (0.91)^a^	2.4 (0.78)^a^	2.37 (0.81)^a^
**My family relationships would be**: 1 = great deal better, 5 = great deal worse	**2.37** (0.89)^**a**^	**2.73 (0.62)** ^**b**^	2.67 (0.68)^b^
My friendships would be: 1 = great deal better, 5 = great deal worse	2.54 (0.81)^a^	2.72 (0.67)^a^	2.73 (0.63)^a^
**My sex life would be**: 1 = great deal better, 5 = great deal worse	**2.28 (**0.9)^**a**^	**2.69 (0.63)** ^**b**^	2.36 (0.89)^a^
**My worries about the future of my family and close friends (e.g. their health, independence, income) would be**: 1 = decrease a great deal, 5 = increase a great deal	**2.25 (0.99)** ^**a**^	**2.55 (0.88)** ^**b**^	2.7 (0.79)^b^
**My motivation to achieve things would be**: 1 = great deal better, 5 = great deal worse	**1.96 (0.88)** ^**a**^	**2.25 (0.93)** ^**b**^	2.12 (0.81)^ab^
The things I could do physically would: 1 = increase a great deal 5 = decrease a great deal	1.86 (0.9)^a^	2.13 (0.87)^a^	2.08 (0.88)^a^
**My quality of life would be**: 1 = great deal better, 5 = great deal worse	**1.70 (0.86)** ^**a**^	**1.99 (0.84)** ^**b**^	1.94 (0.84)^ab^

Different superscript letters next to the data values indicate significant differences between the groups. Values with the same letter superscript indicate no statistical difference

Table 3 depicts the Health Service Measures Across Generational Status. There were no differences in the number of clinic, emergency department, or hospital visits per year across generations. There are no differences in the number of total or diabetic medications. Second generation participants had a statistically higher A1C and BMI than the 1st generation. There were more myocardial infarctions in the 3rd generation than in the other generations.

**Table 3:** Health service measures across generational status

Variable	1st generation	2nd generation	3rd generation
	Mean (SD)	Mean (SD)	Mean (SD)
In past year, mean number of:			
Clinic visits	7.01 (5.51)^a^	6.87 (5.66)^a^	6.22 (3.85)^a^
ER visits	1.07 (2.46)^a^	0.69 (1.58)^a^	0.58 (1.01)^a^
Hospital visits	0.51 (1.17)^a^	0.43 (1.18)^a^	0.27 (0.82)^a^
Number of diabetic meds	2.07 (2.02)^a^	2.07 (1.08)^a^	1.91 (1.25)^a^
Total number of meds	10.83 (10.83)^a^	9.99 (5.22)^a^	10.51 (5.25)^a^
**A1C**	**7.73 (1.73)** ^**a**^	**8.56 (1.93)** ^**b**^	8.10 (1.88)^ab^
**BMI**	**31.69 (7.37)** ^**a**^	**34.95 (7.78)** ^**b**^	34.09 (7.37)^ab^
	Percent	Percent	Percent
**Myocardial Infarction**	0% ^a^	0% ^a^	**2%** ^**b**^
Amputations	4.3% ^a^	2.9% ^a^	6.0% ^a^
Dialysis	7.1% ^a^	4.6% ^a^	2.7% ^a^
Taking Insulin	46.5% ^a^	44.1% ^a^	40.9% ^a^

Different superscript letters next to the data values indicate significant differences between the groups. Values with the same letter superscript indicate no statistical difference

Table 4 shows the QoL across generational status stratified by CI. For those with CI, first generation scores for QoL were significantly lower for the *friendships*, *family relationships*, *worries about the future of family/friends, and sex life* QoL items. Among those with CI, this indicates that the items reflecting concern for connectedness and well-being of family/friends would be improved if they did not have diabetes.

**Table 4:** Quality of Life across generational status by CI

	Chemically Intolerant			Not Chemically Intolerant		
	1st generation	2nd generation	3rd generation	1st generation	2nd generation	3rd generation
	Mean (SD)	Mean (SD)	Mean (SD)	Mean (SD)	Mean (SD)	Mean (SD)
Chemically Intolerant						
**In general, my quality of life is**: 1 = very good, 5 = very bad	2.28 (0.33)^a^	1.75 (0.68)^b^	1.97 (0.72) ^ab^	2.06 (0.74)	1.91 (0.86)	2.05 (0.79)
**If I did not have diabetes**:						
My employment/career opportunities would be: 1 = great deal better, 5 = great deal worse	1.78 (0.88)	2.75 (0.50)	2.36 (0.81)	2.21 (0.9)	2.39 (0.86)	2.33 (0.85)
My worries about my future (e.g., health, independence, income) would: 1 = decrease a great deal, 5 = increase a great deal	2.33 (1.14)	2.31 (1.08)	2.50 (1.05)	2.22 (1.07)	2.5 (0.96)	2.45 (0.91)
My social life would be: 1 = great deal better, 5 = great deal worse	2.24 (0.9)	2.25 (0.93)	2.29 (0.87)	2.14 (0.93)	2.46 (0.72)	2.39 (0.8)
My family relationships would be: 1 = great deal better, 5 = great deal worse	**2.28** **(0.96)** ^**a**^	**2.81** **(0.54)** ^**b**^	2.71 (0.72)^ab^	2.4 (0.88)	2.67 (0.67)	2.64 (0.69)
My friendships would be: 1 = great deal better, 5 = great deal worse	**2.44 (0.92)** ^**a**^	**2.94** **(0.44)** ^**b**^	2.79 (0.59)^ab^	2.58 (0.78)	2.61 (0.74)	2.71 (0.65)
My sex life would be: 1 = great deal better, 5 = great deal worse	**2.12 (0.99)** ^**a**^	**2.88 (0.50)** ^**b**^	2.34 (0.97)^ab^	**2.33 (0.88)** ^**a**^	**2.67 (0.63)** ^**b**^	2.38 (0.86)^ab^
My worries about the future of my family and close friends (e.g., their health, independence, income) would be: 1 = decrease a great deal, 5 = increase a great deal	2.28 (0.96)^a^	2.69 (1.01)^ab^	**2.88 (0.81)** ^**b**^	2.22 (1.03)	2.5 (0.84)	2.64 (0.74)
My motivation to achieve things would be: 1 = great deal better, 5 = great deal worse	1.94 (0.94)	2.25 (1.00)	1.91 (0.83)	1.94 (0.88)	2.26 (0.93)	2.21 (0.79)
The things I could do physically would: 1 = increase a great deal 5 = decrease a great deal	1.83 (0.86)	2.06 (0.85)	2.00 (0.95)	**1.84 (0.92)** ^**a**^	**2.20 (0.88)** ^**b**^	2.08 (0.86)^ab^
My quality of life would be: 1 = great deal better, 5 = great deal worse	1.61 (0.85)	1.88 (0.72)	1.82 (0.83)	1.69 (0.87)	1.96 (0.89)	1.97 (0.84)

Different superscript letters next to the data values indicate significant differences between the groups. Values with the same letter superscript indicate no statistical difference

Among those without CI, relative to 2nd the generation, first generation scores were significantly lower on the *Physical* and *Sex lives* QoL items—indicating that the things they can do physically would be improved if it were not for their Diabetes.

## Discussion

### Diabetes and QoL

The extant literature suggests that Latinos show poorer self-management of diabetes and are disproportionately impacted by diabetes-related complications relative to non-Latino whites [35]. Within the Latino population, immigrants with diabetes are less likely to effectively manage diabetes and meet targets than their U.S.-born counterparts [36]. This finding was not replicated in our study, as immigrants in our study had a lower A1C and BMI relative to 2nd generation participants.

Among Latinos, barriers to effective management and care include multiple social determinants of health, such as housing instability, poverty, and lack of insurance or being underinsured [37]. Additionally, food security has been identified as a significant barrier to the effective management of diabetes. Osborn and colleagues [38] reported immigrant Latinos with food insecurity were less likely to report proper management of diabetes than U.S.-born Latinos. Although housing instability and adequacy of access and insurance were not assessed, our immigrant sample did report lower income than the U.S.-born samples, which suggests additional factors can be at play.

The role of family support in management of diabetes is an important consideration. In a study investigating the effectiveness of an intervention for treatment adherence and weight in Latinos with type 2 diabetes, having friends and family provide instrumental support was associated with treatment adherence and weight loss [39]. The extent to which participants in our study benefit from support from friends and family was not assessed. Further, even when friends and family want to provide support, they might not feel equipped to provide this support given their perceived lack of knowledge [36]. It is possible that immigrants in our study benefitted from extensive friend and family support that contributed to more effective management of diabetes relative to U.S.-born participants. *Familismo*, a core value that refers to the strong identification and attachment to the family, including extended family [40], is associated with better self-management of diabetes among Latinos [41].

Regarding QoL, immigrant Latinos were more likely than U.S.-born Latinos to report that were it not for diabetes, worries about the future of family and close friends (e.g., their health, independence, income) would decrease and their family relationships would improve. Family members of an individual with diabetes experience significant burden and worry [42], an impact of which participants in our study are likely aware. Given the value the many Latino communities place on family, it would be reasonable to consider that immigrant Latinos might perceive they are being a burden and inconveniencing the family. The extent to which Latinos in our study are acculturated to the dominant culture was not assessed. As such, it is unclear whether U.S.-born Latinos adhere to these values to the same extent, and how these might contribute to self-management of diabetes. Nonetheless, these findings point to the importance of family and other kin among immigrant Latinos.

### CI and diabetes quality of life

Given the literature demonstrating greater exposure to toxicants and pollutants among individuals with lower income and communities of color, we expected the immigrant sample in our study would be more likely to endorse items on the BREESI, particularly exposure to chemicals, suggesting a greater risk of CI. Immigrant participants reported lower income than U.S.-born participants. However, individuals in our study who immigrated to the U.S. less likely to have BREESI sores highly suggestive of CI as compared to either of their U.S.-born counterparts (i.e., second- and third-generation) and less likely to endorse problems associated with exposure to chemicals. The fact that an inverse relationship has been demonstrated between income and CI, this finding was not apparent across generational status, which varied by income. This may suggest that there are greater nuances to the experience of CI other than income and will require further investigation.

Of note, our sample consists of patients receiving routine outpatient care from a primary care physician at RRNeT residency clinics. The RRNeT group is geographically diverse and includes family medicine residency programs in rural, urban, and suburban areas in the region. When assessing risk of CI, considering geography might allow for greater understanding of risk of CI. Milojevic and colleagues [43] found concentrations of noxious particulate fractions were higher in areas of greater socioeconomic deprivation. However, relationships between pollution level and socioeconomic deprivation varied by urban-rural status.

As of the writing of this paper, there is no literature investigating CI within a sample of individuals with a chronic metabolic condition, specifically, type 2 diabetes. Although inconclusive, some preliminary evidence suggests that type 2 diabetes shares similarities with autoimmune disorders [44]. Similarly, the literature suggesting that CI is an autoimmune process remains inconclusive. Nonetheless, further investigating how these two conditions interact with each other might further our understanding of risk of CI.

CI and its impact on well-being and functioning among those affected have been studied extensively. However, the literature on how CI affects individuals with other chronic diseases is scarce. Our findings suggest that among individuals with diabetes, CI differentially impacts QoL across generational status.

When compared to their U.S.-born counterparts, immigrant participants in our study with CI were more likely to perceive that aspects related to connectedness and well-being would be improved if they did not have diabetes. Among those without CI, the emphasis was on their ability to engage in physical activities; immigrants perceived this aspect of their lives would improve significantly if they did not have diabetes. That immigrants with CI expressed greater perceived improvement in activities that pertain to connectedness highlights a potential interaction between CI and diabetes and an individual’s ability to connect/interact with family and friends. Interestingly, this finding parallels the finding described above that immigrant with diabetes indicated aspects of their lives that involve friends and family and their well-being would improve were it not for diabetes. Like diabetes, social support plays a significant role in management of CI. Nordin and colleagues found reliance on social support to be an important factor in management of CI, with emotional support being relied on to a larger extent than instrumental and informative support [45]. In a qualitative unpublished study conducted by some of the authors of the present study, some participants reported that CI had a significant impact on their relationships. It is possible that among individuals who are dealing with a chronic disease like diabetes, in addition to CI, perceive a greater strain on their relationships. Those without CI did not express the same concern and instead indicated the ability to physically engage in activities would be most improved, perhaps because these participants are not impacted by the physical and emotional consequences of CI.

### Limitations

The extent to which our findings can be generalized to the general population is a limitation of this study. Some of the clinics from which participants were recruited serve a medically and psychosocially complex patient population. Not only does this limit the generalizability, but it also creates confounds. The present study did not account for the role of other common medical comorbidities (e.g., chronic kidney disease, hypertension, chronic obstructive pulmonary disease) or psychiatric comorbidities common in this patient population. Moreover, controlling for social variables, including housing stability, employment status, and additional indices of socioeconomic status would allow for a greater understanding of the interplay of CI and diabetes.

Additionally, CI was determined by item endorsement on a brief screener, the BREESI. Although this questionnaire has high sensitivity, it is not as specific as the longer, original tool, the Quick Environmental Exposure and Sensitivity Inventory (QEESI). The use of a tool such as the QEESI would not only decrease the risk of including false positives in our study analyses but of shedding light on other relationships, including the impact of CI on functioning.

## Conclusions

As of the time of the writing this paper, the literature exploring the interplay between CI and other chronic conditions, such as type 2 diabetes is scarce. Given the impact of diabetes on quality of life and distress associated with management of diabetes, a greater understanding of the role of other chronic conditions is critical given the social determinants of health that contribute to the experience of both conditions. Additionally, this study highlights generational differences in the impact of diabetes on quality of life, although these were not evident in CI.

## Footnotes

1 Superfund sites are contaminated sites due to hazardous waste dumped, left out in the open, or improperly managed that the Environmental Protection Agency has designated as requiring a long-term response to clean-up of hazardous contaminants.
